# Occurrence of Portal Hypertension and Its Clinical Course in Patients With Molecularly Confirmed Autosomal Recessive Polycystic Kidney Disease (ARPKD)

**DOI:** 10.3389/fped.2020.591379

**Published:** 2020-11-12

**Authors:** Dorota Wicher, Ryszard Grenda, Mikołaj Teisseyre, Marek Szymczak, Paulina Halat-Wolska, Dorota Jurkiewicz, Max Christoph Liebau, Elżbieta Ciara, Małgorzata Rydzanicz, Joanna Kosińska, Krystyna Chrzanowska, Irena Jankowska

**Affiliations:** ^1^Department of Medical Genetics, The Children's Memorial Health Institute, Warsaw, Poland; ^2^Department of Nephrology, Kidney Transplantation and Arterial Hypertension, The Children's Memorial Health Institute, Warsaw, Poland; ^3^Department of Gastroenterology, Hepatology, Feeding Disorders and Pediatrics, The Children's Memorial Health Institute, Warsaw, Poland; ^4^Department of Pediatric Surgery and Organ Transplantation, The Children's Memorial Health Institute, Warsaw, Poland; ^5^Department of Pediatrics and Center for Molecular Medicine Cologne, Faculty of Medicine and University Hospital Cologne, University of Cologne, Cologne, Germany; ^6^Department of Medical Genetics, Center for Biostructure Research First Faculty of Medicine, Medical University of Warsaw, Warsaw, Poland

**Keywords:** autosomal recessive polycystic kidney disease, liver fibrosis, portal hypertension, molecular confirmation, transient elastography, endoscopic variceal ligation

## Abstract

**Purpose:** Liver involvement in autosomal recessive polycystic kidney disease (ARPKD) leads to the development of portal hypertension and its complications. The aim of this study was to analyze the occurrence of the portal hypertension and its clinical course and the dynamics in patients with molecularly confirmed ARPKD in a large Polish center. Moreover, the available options in diagnostics, prevention and management of portal hypertension in ARPKD will be discussed.

**Materials and Methods:** The study group consisted of 17 patients aged 2.5–42 years. All patients had ARPKD diagnosis confirmed by molecular tests. Retrospective analysis included laboratory tests, ultrasound and endoscopic examinations, transient elastography and clinical evaluation.

**Results:** Any symptom of portal hypertension was established in 71% of patients. Hypersplenism, splenomegaly, decreased portal flow and esophageal varices were found in 47, 59, 56, and 92% of patients, respectively. Gastrointestinal bleeding occurred in four of 17 patients. Endoscopic variceal ligation (EVL) was performed at least once in nine patients with esophageal varices.

**Conclusions:** Portal hypertension and its complications are present in a significant percentage of ARPKD patients. They should be under the care of multidisciplinary nephrology-gastroenterology/hepatology team. Complications of portal hypertension may occur early in life. Endoscopic methods of preventing gastroesophageal bleeding, such as endoscopic variceal ligation, are effective and surgical techniques, including liver transplantation, are required rarely.

## Introduction

Autosomal recessive polycystic kidney disease (ARPKD) is a severe hepatorenal disease that is grouped to the ciliopathies—diseases caused by dysfunction of genes encoding primary cilia proteins.

Abnormal structure and/or function of primary cilia results in abnormal embryogenesis of bile ducts, which may lead to a spectrum of disorders including congenital hepatic fibrosis (CHF), Caroli disease or Caroli syndrome (1). At the same time, ciliary pathology entails the development of multiple cysts in both kidneys, which results, depending on the phenotype, in the end-stage renal failure with an early or late clinical manifestation (2).

The most common hepatic manifestation of ARPKD is the congenial hepatic fibrosis (CHF), and less frequently the Caroli syndrome.

Hepatic fibrosis is a progressive process and it entails the development of portal hypertension along with its complications with time.

Portal hypertension (PH) is defined as a pathological increase in pressure within the portal system. The normal pressure in the portal vein is 5–10 mmHg, and the pressure gradient between the portal vein and the inferior vena cava should not exceed 4 mmHg. The portal pressure maintained above 10 mmHg is accompanied by the formation of esophageal varices, whilst the values above 12 mmHg by the ascites and the esophageal varices bleeding (3).

Direct pressure measurement in the portal system requires hepatic vein catheterization, which being highly invasive, is not used in everyday clinical practice.

Indirect methods of the portal hypertension diagnosis are based on an analysis of laboratory parameters (e.g., blood count with thrombocytopenia as the sign of hypersplenism), the results of imaging tests, including an ultrasound examination of the abdominal cavity, which enables to measure the spleen size and a quantitative assessment of portal blood flow with Doppler technique. The endoscopy of the gastrointestinal tract may show esophageal and less frequently gastric and/or anorectal varices.

The aim of this study was to a retrospectively describe the occurrence of the PH and its clinical course and dynamics in a group of patients with ARPKD and confirmed pathogenic variants in the *PKHD1* gene from a large Polish center.

## Patients and Methods

The study group included patients with ARPKD diagnosis confirmed molecularly.

Sanger sequencing of *PKHD1* gene was performed in 10 patients and next generation sequencing (NGS) targeted to the panel of genes associated with polycystic kidney diseases in 7. The NGS analysis was carried out under the resources of M15/16 grant.

Retrospective analysis of laboratory test results and clinical evaluation included:

- evaluation of hypersplenism (thrombocytopenia < 100,000 platelets/μl);
- ultrasound imaging for splenomegaly [spleen size beyond normal values for a given age (4)], presence of the collateral circulation vessels, measurement of portal vein diameter and the flow velocity in the portal system;
- endoscopic examination for esophageal/gastric varices, portal gastropathy, stigmata of life-threatening bleeding (red wale signs—red patches or strips on the varices) and endoscopic procedures;
- transient elastography (FibroScan® suite from Echosens) with evaluation of liver fibrosis.

Information on the past episodes of gastrointestinal tract bleeding and the age of occurrence of particular symptoms associated with PH was recorded.

All the methods were carried out in accordance with relevant guidelines and regulations.

The study protocol was approved by local Bioethical Committee. An informed consent was obtained from all the involved participants.

## Results

The study group included 17 patients with ARPKD at mean age of 2.5–42 years (median: 15 years).

In 4 patients (P2, P8, P9, P16) renal abnormalities were present in prenatal period, in all 17 patients ARPKD was suspected before the age of 5. One patient (P2) with an additional diagnosis of genetically determined cardiomyopathy died at the age of 29 months. In the remaining patients the mean follow-up time after the initial diagnosis was 14 years (6–37 y.).

The characteristic of the cohort (gender, age, time point of diagnosis, family history and renal outcome) is presented in Table 1.

**Table 1:** The characteristic of study group.

**Patient no., sex**	**Age (years)**	**Time point of diagnosis**	**Family history**	**Renal outcome**
P1, M	15	3 months	Negative, no siblings	Progressive kidney disease, RRT considered
P2, F	2.5	Prenatal	Negative, one healthy sister	Unilateral nephrectomy; dialysis; death at the age of 29 months
P3, M	19	3 years	Negative, two healthy sisters	Progressive kidney disease, peritoneal dialysis considered
P4, F	15	1 month	Negative, no siblings	Normal renal function
P5, M	13	3 years	Positive, affected younger sister^*^	Normal renal function
P6, M	9	3 days	Positive, affected younger sister^*^	Slowly progressive kidney disease, without RRT
P7, M	15	1.5 years	Negative, one healthy brother	Normal renal function
P8, M	8	Prenatal	Positive, death of first child (Potter sequence); healthy twin brother	Progressive kidney disease, without RRT
P9, M	9	Prenatal	Negative, one healthy sibling	KTx at the age of 3
P10, M	42	4 years	Positive, death of three affected siblings	Dialysis from 29 years of life,CKLTx at the age of 37 years
P11, M	19	1 month	Negative, two healthy older sisters	Progressive kidney disease, without RRT
P12, F	24	1 month	Negative, one healthy sister	Slowly progressive kidney disease, without RRT
P13, M	19	4 months	No data	Normal renal function
P14, M	42	5 years	Negative, one healthy sister, two healthy children	Dialysis from 40 years of life, considered KTx or CKLTx
P15, M	10	2 years	Negative, no siblings	Normal renal function
P16, M	19	Prenatal	No data	Progressive renal disease, without RRT
P17, M	20	1 year	No data	Progressive renal disease, without RRT

*CKLTx, combined kidney and liver transplantation; F, female; KTx, kidney transplantation; M, male; P, patient; RRT, renal replacement therapy*.

* *not confirmed molecularly yet*.

Molecular analysis showed the presence of biallelic pathogenic/likely pathogenic variants in the *PKHD1* gene in all 17 patients. Molecular variant c.107C>T, p.(Thr36Met) was the most frequent and it was identified in 9/17 patients. Variants c.274C>T, p.(Arg92Trp); c.3364G>A, p.(Gly1122Ser) and c.8114del, p.(Gly2705Valfs^*^11) were repeatedly observed in three unrelated patients (each one), whereas the remaining molecular variants were found only in single patients.

The results of genetic testing are presented in Table 2.

**Table 2:** Molecular variants in *PKHD1* and phenotype in presented patients.

**Patient no., sex**	**Variant 1 nucleotide and amino acid change [accession number in ClinVar]**	**Variant 2 nucleotide and amino acid change [accession number in ClinVar]**	**Phenotype**
P1, M	**c.2747A>C** **p.(His916Pro)** [VCV000978794]	**c.3364G>A** **p.(Gly1122Ser)** [VCV000978797]	Splenomegaly, decreased portal vein flow, hypersplenism, esophageal and gastric varices
P2, F	**c.5895dupA** p.(Leu1966Thrfs*4) [VCV000167486]	**c.9370C>T** **p.(His3124Tyr)** [VCV000555142]	Splenomegaly, decreased portal vein flow, esophageal and ectopic varices, gastrointestinal bleeding
P3, M	**c.107C>T** **p.(Thr36Met)** [VCV000004108]	c.2170C>T p.(Pro724Ser) [VCV000978796]	Splenomegaly, portosystemic collateral pathways, hypersplenism, esophageal and gastric varices, gastrointestinal bleeding
P4, F	**c.107C>T** **p.(Thr36Met)** [VCV000004108]	**c.8114del** p.(Gly2705Valfs*11) [VCV000871259]	Decreased portal vein flow, portosystemic collateral pathways, esophageal varices
P5, M	c.11237T>C p.(Leu3746Pro) [VCV000978804]	**c.8411T>A** **p.(Met2804Lys)** [VCV000197958]	Splenomegaly, decreased portal vein flow, hypersplenism, esophageal and gastric varices
P6, M	**c.107C>T** **p.(Thr36Met)** [VCV000004108]	**c.8114del** p.(Gly2705Valfs*11) [VCV000871259]	Decreased portal vein flow, portosystemic collateral pathways
P7, M	**c.107C>T** **p.(Thr36Met)** [VCV000004108]	c.1001T>A p.(Val334Asp) [VCV000978795]	No clinical signs of portal hypertension
P8, M	**c.107C>T** **p.(Thr36Met)** [VCV000004108]	**c.107C>T** **p.(Thr36Met)** [VCV000004108]	Splenomegaly, portosystemic collateral pathways, hypersplenism, esophageal and gastric varices
P9, M	**c.107C>T** **p.(Thr36Met)** [VCV000004108]	c.3287T>C p.(Leu1096Pro) [VCV000978783]	No clinical signs of portal hypertension
P10, M	**c.107C>T** **p.(Thr36Met)** [VCV000004108]	c.7721T>G p.(Met2574Arg) [VCV000978784]	Splenomegaly, decreased portal vein flow, hypersplenism, esophageal varices
P11, M	**c.274C>T** **p.(Arg92Trp)** [VCV000286684]	**c.3364G>A** **p.(Gly1122Ser)** [VCV000978797]	Splenomegaly, decreased portal vein flow, hypersplenism, esophageal and gastric varices
P12, F	**c.107C>T** **p.(Thr36Met)** [VCV000004108]	**c.8114del** p.(Gly2705Valfs*11) [VCV000871259]	Splenomegaly, decreased portal vein flow, portosystemic collateral pathways, hypersplenism, esophageal and gastric varices, gastrointestinal bleeding
P13, M	c.390+1G>A p.? [VCV000978798]	**c.5498C>T** **p.(Ser1833Leu)** [VCV000496898]	No clinical signs of portal hypertension
P14, M	**c.274C>T** **p.(Arg92Trp)** [VCV000286684]	**c.3364G>A** **p.(Gly1122Ser)** [VCV000978797]	Splenomegaly, hypersplenism, esophageal varices
P15, M	c.4209_4232del p.(Gly1404_Gly1411del) [VCV000978785]	**c.11074C>T** p.(Arg3692*) [VCV000593691]	Splenomegaly, decreased portal vein flow, portosystemic collateral pathways, esophageal varices, gastrointestinal bleeding
P16, M	**c.107C>T** **p.(Thr36Met)** [VCV000004108]	**c.274C>T** **p.(Arg92Trp)** [VCV000286684]	No clinical signs of portal
P17, M	c.880+1G>A p.? [VCV000978787]	**c.4870C>T** **p.(Arg1624Trp)** [VCV000188369]	No clinical signs of portal hypertension

*F, female; M, male; Mutation numbering was based on the human PKHD1cDNA sequence of the GenBank accession no. NM_138694.3; NP_619639.3, the nucleotide position at the A of the ATG translation start codon is denoted as nucleotide +1; **boldface print** - variants known in Human Gene Mutation Database Professional 2019.1 (HGMD, www.hgmd.cf.ac.uk)*.

In five patients (P7, P9, P13, P16, P17), aged 9–20 years, the clinical signs of portal hypertension were not present.

Hypersplenism (defined as thrombocytopenia with or without leukopenia) was found in 8 patients and splenomegaly in 10 (47 and 59%, respectively). Splenomegaly preceded hypersplenism (median age: 5 vs. 6 years; Table 3).

**Table 3:** Occurrence frequency of particular symptoms in the study group and the age at which they were found.

	**Number of patients (%)**	**Age of onset in years (median)**
Hypersplenism^*^	8/17 (47)	2–4 (6)
Splenomegaly	10/17 (59)	1–11 (5)
Collateral circulation vessels	6/16 (38)	0,5–5 (2,5)
Portal flow <16 cm/s	9/16 (56)	1–18 (5)
Liver fibrosis (FibroScan)	7/8 (88)	-
EGD (esophagogastroduodenoscopy)	12/17 (71)	1,5–18 (3)
Esophageal varices	11/12 (92)	1,5–9 (3)
Gastric varices	6/10 (60)	3–9 (7,5)
Portal gastropathy	9/10 (90)	1,5–11 (7)
Stigmata of life-threatening bleeding	3/10 (30)	6–13 (8)
EVL (endoscopic variceal ligation)	9/10 (90)	2,5–13 (8,5)
Gastrointestinal bleeding	4/17 (24)	1,5–13 (8)

* *defined as thrombocytopenia (<100,000 platelets/μl) with or without leukopenia*.

Collateral circulation vessels was described in 38% of patients and cavernous transformation of the portal vein was found in two (P6, P15).

The value of portal vein diameter exceeded normal pediatric values (3–11.2 mm) (5) just in a single case of our group. Decreased portal flow (<16 cm/s) was found in 9/16 respondents (56%).

Esophagogastroduodenoscopy (EGD) was performed in 71% (12/17) of patients. The youngest patient who underwent such examination was 18-months-old and the median age at the moment of the first EGD was 3 years. Esophageal varices were found in 11 (92%), gastric varices in 6 (50%) and ectopic varices in the antrum region in one. The time point of detection of esophageal and gastric varices was 18 months and 3 years of life respectively (Table 3). Portal gastropathy and stigmata of life-threatening bleeding were found in nine and three subjects respectively at the median age of 7 and 8 years.

Esophageal varices were present in all patients with hypersplenism and in all patients with splenomegaly, while hypersplenism was not detected in three patients with esophageal varices and splenomegaly in one. Esophageal varices were found in only one out of seven patients with a normal portal flow. In a subgroup of patients with a decreased portal flow endoscopic features of PH were present in eight out of nine patients (89%).

Gastrointestinal tract bleeding was observed in four patients (24%), aged 1.5–13 years (median: 8 years), In three of them twice (in two subjects it occurred within the same year and in one of the subject at an interval of 7 years). EVL was performed in nine patients (in four after bleeding, in five as preventive therapy) and was repeated in five (two to five times). Median age of first EVL was 8.5 years. Four patients underwent sclerotherapy or local administration of the tissue adhesive. In two patients gastrointestinal tract bleeding was indication for first EGD.

In eight patients transient elastography (Fibroscan) was performed. Normal result was seen only in one who did not show any signs of PH. In the remaining patients liver fibrosis was detected (12.3–75 kPa). Endoscopic features of PH were found in six out of seven patients with abnormal results of the transient elastography.

In our group nine received propranolol (dose ranged from 0.5 to 2 mg/kg/day), five for arterial hypertension and four patients to lower portal system pressure. In the former subgroup esophageal varices were detected in three patients aged 1, 2, and 9 years, respectively, and gastrointestinal tract bleeding was additionally found in one patient at the age of 18 months. On the other hand esophageal varices were detected in all patients before the pharmacotherapy; in the subsequent years preventive endoscopic procedures were performed in all patients, therefore effect of propranolol on PH was difficult to assess.

Only one patient received kidney transplant at the age of 2. During 7 years of the follow-up post-transplant he did not show any signs of PH or liver complications. Another patient was dialyzed from the age of 29 years and at the age of 38 years he underwent combined kidney and liver transplantation (CKLTx).

## Discussion

Primarily, the CHF on the microscopic level was found in all ARPKD patients, while clinical symptoms gradually appeared over time as the fibrosis progressed (6–8).

In order to detect liver-related complications, spleen assessment on physical examination, annual complete blood and platelet count, as well as abdominal ultrasound at 5 years of life (if negative, it should be repeated every 2–3 years) are recommended (9).

At least one symptom of PH: hypersplenism, splenomegaly, decreased portal flow or endoscopic features may be present in 37–68% of patients with ARPKD (8–12), while was seen in 71% of our patients. No correlation between PH and renal failure was observed, which is consistent with other reports (8). We did not observe significant genotype-phenotype correlations in the analyzed group, probably due to the relatively small number of patients. The most common *PKHD1* variant c.107C>T, p. (Thr36Met) was detected both in patients with PH and in patients with no evident features of liver involvement.

The threshold number of platelets, at which thrombocytopenia was diagnosed in children, is not uniform and different values were considered as subnormal (115,000–150,000/μl). The current definition of thrombocytopenia is the platelet count of <100,0000/μl and this was adopted to our analysis. Hypersplenism was observed in 47% of our patients, which is consistent with the other published data (8, 11–13).

USG- proven splenomegaly was reported in 35–65% of patients with ARPKD and in 59% of our patients. Results obtained in our cohort confirmed that decreased portal flow (<16 cm/s) is correlated with PH—endoscopic features of PH were present in 89% of patients with decreased portal flow. Thus, we agree that this is an indication for esophagogastroduodenoscopy.

Reports on use and predictive value of a transient elastography (TE) in evaluation of PH in patients with ARPKD are scarce, however it is regarded as useful in early detection of hepatic fibrosis in ARPKD (14, 15). In presented group PH was detected in all but one patients with abnormal results of TE.

In adults with PH with no history of bleeding from the gastrointestinal tract non-selective β-blockers are used in primary prevention. If the effect is unsatisfactory, vasodilator drugs (nitrates) are co-administered. Limited studies reported beneficial effects of propranolol in children, therefore preventive administration of propranolol in children is not widely recommended (6, 16, 17).

Non-selective β-blockers are sometimes used in pharmacotherapy of hypertension in children, therefore double clinical profit is available. As mentioned in the result section, we were not able to assess the effectiveness of propranolol in our cohort.

The presence of esophageal varices grade III, esophageal varices grade II with stigmata of life-threatening bleeding and/or gastric varices is associated with high risk of gastrointestinal bleeding (18). In the analyzed group “high-risk varices” were found in nine of 10 examined patients and bleeding occurred in four of them (44%). EVL is an effective and safe method to prevent primary and secondary bleeding from esophageal varices and this was confirmed in long-term experience of our center (19). EVL was performed in nine patients (in four after bleeding) and two of them had sclerotherapy and varices obliteration with the tissue adhesive. According to the expert opinion, routine primary prevention should be based on EVL in pediatric patients with high risk of gastrointestinal tract bleeding (20).

EVL is effective in prevention of the esophageal varices bleeding, but it does not reduce the risk of other complications of PH.

Surgical methods used in patients with PH include variety of vascular anastomoses, like mesocaval, portacaval, and splenorenal shunts. These methods can effectively reduce the pressure in the portal system, but might be associated with serious side effects. The best results of vascular anastomosis are observed in patients with PH in the course of prehepatic block; otherwise they may be used as a supportive management until LTx is available. In general, the surgical management of PH in the era of LTx and endoscopic techniques is currently of minor importance.

As preventive endoscopic procedures were effective in our patients, no vascular anastomosis was considered.

Indications for LTx in patients with PH include the recurrent, endoscopically incurable bleedings from esophageal varices and/or liver decompensation. In the cases of ARPKD, recurrent cholangitis may be an additional indication. Similarly, combined or sequential kidney and liver transplantation (CKLTx/SKLTx) may be indicated in patients with end-stage kidney disease due to ARPKD.

There are different transplantation strategies in patients with ARPKD (21). Supporters of isolated kidney transplantation argue that the long-lasting preserved liver function and the efficacy of endoscopic prevention of bleeding from esophageal varices may postpone the need for LTx. On the other hand, immunosuppression after renal transplantation in patients with biliary dilatation may increase the risk of the difficult-to-treat biliary infections, which may cause sepsis (16).

Out of 27 patients with ARPKD presented by Luoto et al. 10 underwent a LTx—in one case isolated liver (in a patient with a suspected hepatoblastoma) and in the remaining ones CKLTx, including five cases of SKLTx (8).

Overall, CKLTx/SKLTx was performed in 15 patients with clinical diagnosis of ARPKD in our center in 1984–2017. They are regarded as historical cohort (22). Out of current series of 17 patients, only two required transplantation (one underwent KTx, other—CKLTx). These data show the effectiveness of modern conservative treatment and endoscopic gastrointestinal procedures, which allow to limit the indication for LTx in children with early detected complications of hepatic fibrosis in the course of ARPKD.

## Conclusions

CHF is a progressive process and it leads to complicated PH over time.

Detection of PH should implement monitoring and treatment of avoidable complications related to high pressure in the portal system. Currently, the most effective method of preventing the bleeding from esophageal varices is EVL. If not effective, patient require use of surgical treatment, including LTx, anyway appropriate conservative management may delay or deny the indication for this procedure. Molecular diagnosis is supportive, however not determining the clinical management.

## Data Availability Statement

All datasets generated for this study are included in the article/supplementary material.

## Ethics Statement

The studies involving human participants were reviewed and approved by Children's Memorial Health Institute Bioethical Committee, Warsaw, Poland. Written informed consent to participate in this study was provided by the participants' legal guardian/next of kin.

## Author Contributions

DW: the conception, design, analysis, interpretation of the data, and the drafting of the paper. IJ and RG: revising paper critically for intellectual content and the final approval of the version to be published. KC, MCL, MS, and MT: revising paper critically for intellectual content. JK, MR, EC, DJ, and PH-W: analysis and interpretation of the data. All authors contributed to the article and approved the submitted version.

## Conflict of Interest

MCL has been counseling Otsuka in an advisory board, is a member of the European Reference Network for Rare Kidney Diseases (ERKNet)– Project ID No 739532, of the Council of the European Society for Pediatric Nephrology, and an associate editor of Frontiers in Pediatrics, Section Pediatric Nephrology. The remaining authors declare that the research was conducted in the absence of any commercial or financial relationships that could be construed as a potential conflict of interest.
